# Pregnancy outcomes among women affected with thalassemia traits

**DOI:** 10.1007/s00404-022-06519-y

**Published:** 2022-03-26

**Authors:** Pornpimol Ruangvutilert, Chayawat Phatihattakorn, Chutima Yaiyiam, Tachjaree Panchalee

**Affiliations:** 1 Division of Maternal-Fetal Medicine, Department of Obstetrics and Gynecology, Faculty of Medicine Siriraj Hospital, Mahidol University, Bangkok, 10700 Thailand; 2 Department of Obstetrics and Gynecology, Faculty of Medicine Siriraj Hospital, Mahidol University, Bangkok, 10700 Thailand

**Keywords:** Thalassemia, Pregnancy outcomes, Thalassemia trait

## Abstract

**Objective:**

To compare the maternal and perinatal outcomes between a group of pregnant women diagnosed with thalassemia traits and normal controls.

**Study design:**

A retrospective cohort study was conducted on singleton pregnant women affected and unaffected by thalassemia traits who attended an antenatal care clinic and delivered in Siriraj Hospital. Thalassemia status for all subjects was diagnosed by hemoglobin typing and/or DNA analysis. Patient charts were reviewed from January 2007 to December 2018. The control participants were randomly selected from the same period, with a control–case ratio of around 1:1.

**Results:**

Overall, 1288 women with thalassemia traits (348 with α thal-1 trait, 424 with β thal trait and 516 with HbE trait) and 1305 women in the control group were recruited. Baseline characteristics of both groups were similar, with the exception that the hematocrit level in the first trimester in the thalassemia trait group was significantly lower than that in the control group (34.8 ± 3.4% VS 36.9 ± 3.0%; *p < *0.001). The prevalence of pregnancy-induced hypertension (PIH) was higher in the thalassemia trait group, at 6.9% VS 4.7% in the control group; *p = *0.018. When subgroups were analyzed between each thalassemia trait, the number of maternal anemias in the first and third trimester was higher for all thalassemia traits compared to the normal group. The β thal and HbE traits increased the risk of PIH, with a relative risk (RR) = 1.67 and 1.66, respectively.

**Conclusions:**

Thalassemia traits minimally but significantly increase the risk of hypertensive disorders and maternal anemia. In addition, physiological changes during pregnancy may worsen the severity of anemia in the pregnant women with thalassemia traits.

## Introduction

Thalassemia is one of the most common monogenic disorders. It affects hemoglobin production and is highly prevalent in Southeast Asia [1]. Hemoglobin is a protein in red blood cells which carries and transfers oxygen to all tissue and organs in the human body [2]. Each hemoglobin molecule comprises a tetramer made of four globin subunits including two types, alpha and beta globin subunits, which are produced by alpha and beta genes, respectively [3]. Therefore, thalassemia disorders depend on which globin gene is non-functioning, and is diagnosed as alpha or beta thalassemia.

Generally, the prevalence of thalassemia carriers in Thailand is approximately 40%. 2.7–3.5% have heterozygous beta thalassemia (β thal trait; β^0^ or β^+^), 21.9–37.5% heterozygous hemoglobin E (HbE trait), 1.8–8.0% homozygous hemoglobin E and 4.5–5.3% heterozygous alpha thalassemia-1 (α thal-1 trait) [4, 5]. To identify thalassemia trait couples who are at risk of having a baby with major thalassemia disease, prenatal thalassemia screening has been established as a national policy for almost 20 years, consisting of complete blood count (CBC) and hemoglobin typing (Hb typing) [1]. In addition, the aim of this strategy is to reduce the incidence of new affected neonatal cases, with termination of pregnancy allowed for severe thalassemia disease. The number of prenatal diagnoses among pregnant women who are at risk of having affected babies has risen tremendously with this screening policy.

Thalassemia is an autosomal recessive disorder, where two copies of an abnormal gene must be present for the disease, while carriers or traits who have one abnormal copy have no symptoms but can transfer the abnormal gene to their offspring. The severity of thalassemia varies according to the number of functioning globin genes. Alpha thalassemia major is described as the loss of all four alpha globin genes (–/–), while loss of three globin genes presents in Hemoglobin H disease (HbH; –/− α). Alpha thal-1 trait has a loss of two functioning alpha globin genes (–/αα) and α thal-2 trait has a loss of only one normal alpha globin gene (- α/αα). [6] In beta thalassemia, patients who lose both beta globin genes (-/-) usually present with symptomatic anemia, but carriers with one normal gene have no symptoms or mild anemia. In addition, a common single point mutation in the beta gene is known as HbE [7].

In general, α thal-1, β thal and HbE traits present with an abnormal CBC (microcytic hypochromic RBC), without significant clinical symptoms. Thalassemia traits are usually diagnosed among couples who seek pre-conceptional carrier screening or during antenatal screening for thalassemia diseases. According to several studies, pregnant women with thalassemia traits are at risk of adverse pregnancy outcomes, including preterm labor, intrauterine growth restriction and low birth weight [8–12]. However, based on our current knowledge, the effects of thalassemia traits on other obstetric complications and their pathophysiology are still unclear. Although, thalassemia trait women are normally asymptomatic before pregnancy, physiological changes during pregnancy (30% increase in red blood cell volume and 40% increase in total blood volume) can deteriorate leading to anemia during pregnancy (13).

Given the high prevalence of thalassemia in Thailand and a lack of a consensus on the effect of thalassemia traits on pregnancy outcomes, the objective of this study was to compare maternal and neonatal outcomes between women with thalassemia traits and a control group of pregnant women. We also aimed to carry out a subgroup analysis among each type of thalassemia carrier and determine the risk ratio for adverse pregnancy outcomes in pregnant women who are complicated by thalassemia traits.

## Materials and methods

This retrospective cohort study was approved by the Siriraj Institutional Review Board. The study was conducted at the Department of Obstetrics and Gynecology, Faculty of Medicine Siriraj Hospital, Mahidol University, Thailand. Faculty of Medicine Siriraj Hospital is the biggest hospital and the referral tertiary center in Thailand. Patient charts were reviewed between January 2007 and December 2018, and were recorded in the Thalassemia Antenatal Care Clinic and Obstetrics In-patient Ward database. Singleton pregnant women with antenatal care visiting and giving birth at Siriraj Hospital were included. The cases were identified with (1) α thal-1 trait (2) β thal trait or (3) Hb E trait. All pregnant women were undergone the thalassemia screening with CBC and hemoglobin typing. In cases with suspected α thal-1 trait, alpha DNA was confirmed by DNA testing. The control participants were randomly selected in the same period with a case–control ratio of around 1:1, based on similar baseline characteristics of maternal age, maternal body mass index (BMI) and parity. The exclusion criteria were the following; (1) unknown thalassemia status; (2) incomplete clinical data and pregnancy outcomes; (3) affected fetuses with thalassemia major; (4) fetuses with severe structural anomalies or chromosomal abnormalities; and (5) pregnant women with other medical or surgical complications during pregnancy, such as overt diabetes mellitus (DM), chronic hypertension and renal disease.

At the first visit, gestational age and estimated date of delivery were confirmed by both clinical examination and ultrasonography. Maternal blood and urine were tested as for standard antenatal care. Maternal charts were reviewed to collect the demographic data, including maternal age, parity, maternal BMI, gestational age, past history, and laboratory findings. Ultrasonography was carried out for fetal structural examination at mid-pregnancy. Obstetric complications were defined as (1) maternal anemia: hemoglobin (Hb) level < 11 g/dl or hematocrit (Hct) level < 33% [14] (2) pregnancy-induced hypertension (PIH): including gestational hypertension (a systolic blood pressure ≥ 140 mmHg or a diastolic blood pressure ≥ 90 mmHg, or both, on two occasions at least 4 h apart after 20 weeks of gestation in a woman with a previously normal blood pressure), preeclampsia (a new onset of hypertension after 20 weeks of gestation with a new onset of proteinuria or abnormal symptoms/laboratory findings), eclampsia (the convulsive manifestation of the hypertensive disorders of pregnancy) and HELLP syndrome (a more severe form of preeclampsia with hemolysis, elevated liver enzymes, and low platelet count) [15] (3) gestational diabetes mellitus (GDM): diagnosed during the current pregnancy with at least 2 abnormal values from a 100 g oral glucose tolerance test (OGTT) [16] (4) postpartum hemorrhage (PPH): a cumulative blood loss of greater than or equal to 1000 mL [17] (5) mode of delivery: vaginal delivery, cesarean delivery and operative vaginal delivery with forceps or vacuum and (6) preterm delivery: giving birth before completing 37 weeks of gestation [18]. Maternal BMI was defined according to the World Health Organization (WHO) classification: underweight (BMI < 18.5 kg/m^2^), normal weight (BMI 18.5–24.9 kg/m^2^), overweight (BMI 25.0–29.9 kg/m^2^) and obesity (BMI ≥ 30 kg/m^2^).

Neonatal outcomes were reviewed from both maternal and neonatal charts. The variables of interest were the following: (1) low birth weight (LBW): a birth weight < 2,500 g, as per the WHO classification (2) small for gestation age (SGA): a birth weight < 10th percentile for gestation (19) (3) birth asphyxia or low Apgar score: an Apgar score at 1 and 5 min < 7 [20] and (4) still birth: fetal death in utero after completing 20 weeks of gestation. [12] Also, neonatal intensive care unit admission data were collected.

### Statistical analysis

Data were analyzed by the SPSS program, version 18.0 (SPSS statistics for Window, SPSS Inc., Chicago III., USA). The collected data were tested for normality using the Kolmogorov–Smirnov test. Categorical data are shown by number or percentage. Continuous data are calculated and presented as mean ± standard deviation (SD). Maternal and neonatal outcomes were compared between the thalassemia traits and control groups by the chi-square test and t-test for categorical and continuous data, respectively. The comparisons among each thalassemia trait were tested using a chi-square test for categorical data and ANOVA for continuous data. Relative risks (RR), with 95% confidence intervals (CI), were calculated to present the risk ratio for thalassemia traits for adverse pregnancy outcomes. The adjusted odd ratio (OR) was analyzed. A *p* value < *0.05* was considered statistically significant.

## Results

A total of 1288 women with thalassemia traits (348 with α thal-1 trait, 424 with β thal trait and 516 with HbE trait) and 1305 women in the normal control group were recruited into this study. The baseline characteristics of the case and control groups are shown in Table 1. There were no significant differences regarding maternal age, BMI, parity and fetal gender between both groups. The hematocrit level at the first trimester in the thalassemia trait group was significantly lower than that in the control group (34.8 ± 3.4% VS 36.9 ± 3.0%; *p < *0.001). Similarly, the number of anemic pregnant women in the first trimester in the thalassemia trait group was 378/1288 (29.3%) compared to 119/1305 (9.1%) in the control group; *p < *0.001. None of the pregnant women required blood transfusion throughout pregnancy until the postpartum period.

**Table 1:** Baseline characteristics of pregnant women with thalassemia traits and control

Characteristics	Thalassemia traits group (*n* = 1288)	Control group (*n* = 1305)	*p* value
Maternal age (years); mean ± SD	29.3 ± 5.8	29.4 ± 5.7	0.177
Maternal BMI (kg/m^2^); mean ± SD	21.9 ± 3.9	21.7 ± 4.0	0.143
Maternal BMI group; *n* (%)			0.342
< 18.5	226	253	
18.5–24.9	833	849	
25.0–29.9	178	154	
≥ 30.0	51	49	
Parity; *n* (%)			0.968
Nulliparous	733	741	
Multiparous	555	564	
Hematocrit level at the first trimester; mean ± SD	34.8 ± 3.4	36.9 ± 3.0	< 0.001
Anemia at the first trimester; *n* (%)	377	119	< 0.001
Fetal gender; *n* (%)			0.530
Male	627	652	
Female	661	653	

*BMI* body mass index

Regarding the pregnancy outcome (Table 2), 6.9% of pregnant women in the study group developed PIH VS 4.7% of those in the control group (*p = *0.018). The number of pregnant women affected with anemia during the third trimester in the thalassemia trait group was 482/1288 (33.2%), which was significantly higher than 156/1305 (12.0%) in the control group; *p < *0.001. Additionally, thalassemia trait pregnant women gained more weight during pregnancy than those women in the normal group (13.4 ± 6.5 VS 13.9 ± 5.8 kg, *p = *0.048). There were no significant differences between both groups in terms of gestational age at delivery, mode of delivery, estimated blood loss, PPH, GDM and stillbirth.

**Table 2:** Pregnancy outcomes of thalassemia traits and control groups

	Thalassemia traits group (*n* = 1288)	Control group (*n* = 1305)	*p* value
Anemia at the third trimester; *n* (%)	428	156	< 0.001
Gestational age at delivery (weeks); mean ± SD	38.3 ± 1.6	38.3 ± 1.5	0.469
Mode of delivery; *n* (%)			0.060
Spontaneous vaginal	674	650	
Cesarean section	584	637	
Operative vaginal	30	18	
Total weight gain (kg); mean ± SD	13.4 ± 6.5	13.9 ± 5.8	0.048
EBL (mL); mean ± SD	312.37 ± 224.3	327.1 ± 219.9	0.063
PPH; *n* (%)	16	27	0.124
PIH; *n* (%)	89	61	0.018
GDM; *n* (%)	103	93	0.415
Stillbirth; *n* (%)	3	0	0.122

*EBL* estimate blood loss, *PPH* postpartum hemorrhage, *PIH* pregnancy-induced hypertension, *GDM* gestational diabetes mellitus

After excluding three stillbirth cases in the thalassemia group, neonatal outcomes were analyzed and are shown in Table 3. The mean birth weight was not significantly different between the study and control groups (*p = *0.309). Although the rates of preterm birth, SGA, low Apgar score at 5 min and NICU admission tended to increase in the pregnant women from the study group, the difference was not significant when compared to those in the control group. Only 2393 participants who delivered at term pregnancies (37 0/7–42 0/7 weeks) were analyzed for LBW; no significant difference was observed between the two groups. No post-term pregnancies were observed in this study.

**Table 3:** Neonatal outcomes of thalassemia traits and control groups

	Thalassemia traits group (*n* = 1285)	Control group (*n* = 1305)	*p* value
Birth weight (grams); mean ± SD	3048.0 ± 443.4	3069.3 ± 451.6	0.309
Preterm birth; *n* (%)	106	91	0.236
SGA; *n* (%)	111	105	0.619
LBW^a^; *n* (%)	56	69	0.314
Apgar Score < 7 at 5 min; *n* (%)	3	1	0.371
NICU admission; *n* (%)	13	11	0.687

*SGA* small for gestational age, *LBW* low birth weight, *NICU* neonatal intensive care unit

^a^LBW analyzed for term newborn only (1179 in thalassemia traits and 1214 in control group)

The subgroup analysis among different types of thalassemia traits is shown in Table 4 for the significant outcomes, including anemia and PIH. The mean of the hematocrit level during the first trimester was 33.4 ± 3.2%, 35.1 ± 3.3%, 35.7 ± 3.2, and 36.9 ± 3.0% in women with the β thal trait, α thal-1 trait, HbE trait and normal groups, respectively, with a significant difference between each group (*p < *0.001). The number of anemic pregnant women was similar to the hematocrit level, at 46.9%, 25.6%, 17.2%, and 9.1% of the pregnant women from the β thal trait, α thal-1 trait, HbE trait, and normal groups, respectively (*p < *0.001). All thalassemia traits increased the risk of anemia in the third trimester; the β thal trait had the highest risk with RR 4.06 (95% CI = 3.41–4.85). The risk of PIH was higher in the β thal trait (RR = 1.67) and HbE trait groups (RR = 1.66).

**Table 4:** Subgroup analysis of significantly different variables among thalassemia trait groups

	Control (*n* = 1305)	Alpha thal-1 trait (*n* = 348)	Beta thal trait (*n* = 424)	HbE trait (*n* = 516)	*p* value^*^
Hematocrit at the 1st trimester; mean ± SD	36.9 ± 3.0	35.1 ± 3.3	33.4 ± 3.2	35.7 ± 3.2	< 0.001^a^
Anemia at the 1st trimester; *n* (%)	119	89 (25.6%)	199 (46.9%)	89 (17.2%)	< 0.001^a^
RR (95% CI)	1	2.80 (2.19–3.59)^**^	5.15 (4.22–6.28)^**^	1.89 (1.47–2.44)^**^	
Anemia at the third trimester; *n *(%)	156	92 (26.4%)	206 (48.6%)	130 (25.2%)	< 0.001^b^
RR (95% CI)	1	2.21 (1.76–2.78)^**^	4.06 (3.41–4.85)^**^	2.11 (1.71–2.60)^**^	
PIH; *n* (%)	61	16 (4.6%)	33 (7.8%)	40 (7.8%)	0.018^c^
RR (95% CI)	1	0.98 (0.59–1.71)	1.67 (1.11–2.51)^**^	1.66 (1.13–2.44)^**^	

*RR* relative risk, *95% CI* 95% confidence interval, *PIH* pregnancy-induced hypertension

*P value: calculated by chi-square test for categorical data and ANOVA for continuous data

**Statistically significant

^a^The difference was found between each group

^b^The difference was found between each group; except comparison between the alpha trait and HbE trait group

^c^The difference was found only between the normal VS beta thal trait groups, and the normal VS HbE trait groups

Using a multiple logistical regression model of thalassemia traits and maternal anemia in the first trimester as risk factors, an adjusted odds ratio (OR) of β thal trait and HbE trait groups was associated with PIH (Table 5). Maternal anemia in the first trimester was significantly associated with the rate of SGA and LBW; adjusted OR = 1.89 (95% CI = 1.35–2.64) and 1.72 (1.11–2.69), respectively.

**Table 5:** Multiple logistic regression analysis of thalassemia traits and maternal anemia at the first trimester for pregnancy outcomes

	Thalassemia trait; adjusted OR (95% CI)			Anemia; adjusted OR (95% CI)
	Alpha thal-1 trait	Beta trait	HbE trait	
PIH	1.01 (0.58–1.79)	1.85 (1.16–2.96)^*^	1.74 (1.15–2.63)^*^	0.82 (0.52–1.28)
GDM	1.19 (0.76–1.85)	1.10 (0.70–1.72)	1.27 (0.88–1.85)	0.76 (0.40–1.15)
PPH	0.70 (0.26–1.83)	0.57 (0.21–1.56)	0.56 (0.23–1.37)	0.99 (0.42–2.35)
Preterm delivery	1.29 (0.84–2.00)	1.10 (0.70–1.70)	1.35 (0.93–2.00)	1.02 (0.62–1.67)
SGA	1.02 (0.67–1.56)	0.84 (0.55–1.28)	0.96 (0.66–1.40)	1.89 (1.35–2.64)^*^
LBW	0.80 (0.45–1.41)	0.71 (0.41–1.23)	0.70 (0.42–1.17)	1.72 (1.11–2.69)^*^
NICU admission	1.86 (0.67–5.18)	0.90 (0.26–3.07)	0.66 (0.18–2.37)	1.71 (0.66–4.42)

*OR* odd ratio, *95% CI* 95% confidence interval, *PIH* pregnancy-induced hypertension, *GDM* gestational diabetes mellitus, *PPH* postpartum hemorrhage, *SGA* small for gestational age, *LBW* low birth weight, *NICU* neonatal intensive care unit

*Statistically significant

## Discussion

Thalassemia is highly prevalent among the population with Thai and Asian ancestry. According to the annual report of Siriraj Hospital, almost 50% of pregnant women are affected by thalassemia traits, including α thal-1 trait, β thal trait, and HbE trait. The objective of our study was to determine the maternal and neonatal adverse outcomes in pregnant women in the thalassemia trait group compared to those who were not affected by thalassemia. This study contained 1288 cases and 1305 controls, and is one of the largest studies on pregnancy outcomes in pregnant women with Thalassemia traits. This study found the significantly increase the risk of hypertensive disorders and maternal anemia in thalassemia trait women.

Generally, thalassemia traits are asymptomatic or may cause mild microcytic hypochromic anemia [6, 7]. This study showed that the pregnant women affected with thalassemia trait had a lower hematocrit level compared to that of the normal pregnant women. Also, the number of anemic pregnant women at the first and third trimester was significantly larger in the thalassemia trait group. This finding is similar to those from previous studies, and a systematic review which supports the evidence of worsening anemia in women with thalassemia traits during pregnancy [9, 10, 18, 21, 22]. When compared to women who are not complicated by thalassemia, physiological alterations during pregnancy may cause more anemia in thalassemia carriers. This is very likely to be explained as a result of the effect of thalassemia of ineffective erythropoiesis and pre-exited microcytic hypochromic RBCs. As a result of more severe anemia, pregnant women with thalassemia traits would have a higher risk of adverse pregnancy outcomes compared to normal pregnant women. In the subgroup analysis for each thalassemia trait, the number of anemic pregnant women in the β thal trait group was higher when compared to the other groups, which is in line with the lowest hematocrit level in this group. A previous study also found that pregnant women with β thal traits have a higher risk of a combined folate and iron deficiency anemia [22]. These findings indicate that the β thal trait probably leads to more severe anemia due to the abnormal hemoglobin ratio, with a high level of HbA2. Although the number of the anemic pregnant women in the α thal -1 trait and Hb E trait group were lower than that in the β thal trait group, those numbers were significantly higher than in the control group. As a result of more pronounced anemia in the thalassemia traits, the hematocrit level should be closely monitored to maintain blood counts at a normal level.

Several previous studies have concluded that the effect of thalassemia traits on pregnancy outcomes is inconsistent. Some studies have shown that thalassemia traits in pregnant women represent “an uneventful journey”, whereas other have shown that the prevalence of adverse pregnancy outcomes, such as PIH or SGA, increase in pregnant women affected by thalassemia traits [10, 11, 23]. In our study, the thalassemia trait groups have a higher risk of PIH. With subgroup analysis, the incidence of PIH is higher in the β thal trait and HbE trait groups compared to the normal group. In contrast to the α thal-1 trait group, the incidence of PIH is not different when compared to the normal group. Hanprasertpong et al., 2013 also found a higher incidence of preeclampsia in their thalassemia group [10]. Kemthong et al. 2016 found no significant different among HbE trait and adverse pregnancy outcome, but this study did not mention about maternal anemia as same as our study [24]. Nevertheless, a systematic review in 2010 concluded that the relationship between hemoglobinopathy and PIH is unclear [22]. Previous studies have found that the level of inflammatory cytokines such as C-reactive protein (CRP) increases in patients with beta thalassemia intermedia [25]. Several studies have also suggested that preeclampsia is related to the level of inflammatory cytokines, with a positive relationship [26–28]. In addition, some studies have reported acute infarction as a rare condition in beta thalassemia traits and intermedia [29, 30]. Thus, these mechanisms may cause placental insufficiency in both β thal trait and HbE trait pregnant women and lead them to have a higher risk of PIH. There is no such effect with α thal-1 trait.

In addition, a paternal factor may affect the risk of preeclampsia, but there is a limitation in this study; especially in the normal thalassemia cases. Also, there are several possible mechanisms of preeclampsia. The regression analysis on age and parity found that there is no significant different among these variables in our study.

Although total weight gain in thalassemia trait pregnant women was significantly higher than that in the control group (*p = *0.048) in our study, there was no clinical significance because of a small difference. Our study represented the mean BMI in the normal weight range, with adequate total weight gain in both studied groups. In addition, the number of pregnant women in each BMI classification was similar in both groups of pregnant women, with and without thalassemia traits. Therefore, the effect of maternal BMI and weight gain during pregnancy should not alter our results in both the thalassemia trait and normal groups.

We found no statistically significant differences in maternal and perinatal outcomes, gestational age at delivery, mode of delivery, EBL, PPH, GDM, stillbirth, birth weight, preterm birth, SGA, LBW, low Apgar score at 5 min, or NICU admission between the thalassemia trait and control group. Although the systematic review in 2018 demonstrated that maternal anemia in the first trimester of pregnancy can be considered as a risk factor for SGA which is different from the present study. It can be explained that pregnant women in this study were in the normal weight group and had adequate weight gain which is possibly a protective factor of abnormal fetal growth [31]. Likewise, many previous studies have provided evidence that thalassemia traits do not increase these complications [8, 21–23]. Presumably, thalassemia traits do not affect the risk of most adverse pregnancy complications.

Although there are no data about serum iron or iron deficiency anemia, maternal anemia should be taken into account. At the first visit of antenatal care, CBC with Hb/Hct can guide the healthcare providers to give an adequate iron supplement. Several previous studies demonstrated that anemia during pregnancy is associated with adverse pregnancy outcomes, including hypertension, GDM, preterm birth, and SGA, and especially anemia in the first trimester or the first half of pregnancy which is the critical time of placentation [32–34]. Using multiple logistic regression analysis in this study, maternal anemia in the first trimester increased the risk of small babies diagnosed SGA and LBW. Chronic maternal anemia throughout pregnancy represents a common and reversible associated factor with pregnancy maternal and neonatal morbidity and mortality. So, women with thalassemia traits who have more severe anemia may experience worse pregnancy outcomes than those who do not have anemia.

Apart from the adverse pregnancy outcomes, maternal β-thal trait can affect the offspring long-term outcomes. The study in 2021 showed that maternal β-thal trait is independently associated with offspring long-term hematological morbidity (35).

Our study identified the subjects in both groups had similar baseline characteristics, so the other associated factors such as maternal BMI, age, and parity were neutralized. Other potential confounders like fetal anomalies and underlying maternal disease were also excluded. Additionally, all subjects in the study group had a confirmed diagnosis of thalassemia traits of α thal-1 trait, β thal trait, and HbE trait by Hemoglobin typing and/or DNA testing. The number of samples was adequate to achieve the power for differentiating most of the interesting outcomes. Another strength of this study is the high homogeneity of our subjects in a single center. However, there are some limitations in our study. Although we can collect most of the data from the entire charts, there still were a few charts with some missing data due to the retrospective nature of the study. Furthermore, the small sample size for low Apgar score at 5 min and NICU admissions might not be sufficient to distinguish a difference between the two groups. Further prospective studies should be considered to confirm these results.

According to the guideline for prenatal screening in Thailand and the American College of Obstetrics and Gynecology, a CBC with RBC indices should be performed in all pregnant women to assess not only their anemic status, but also to allow for assessment of the risk of hemoglobinopathy, including thalassemia. If possible, carrier screening is recommended before conceiving. Hemoglobin electrophoresis should be performed in addition to a CBC if thalassemia is suspected based on ethnicity (Southeast Asia, West Indian, African, Mediterranean, and Middle Eastern descent) [36]. Beta thalassemia trait and HbE trait can be diagnosed with a CBC and Hb electrophoresis, with a low mean corpuscular volume (MCV) and high HbA2 or HbE level. In the case of a low MCV but normal Hb electrophoresis, DNA testing should be analyzed for abnormal alpha globin genes. Close monitoring of thalassemia traits among pregnant women, along with adequate folate and iron supplementation, can prevent chronic anemia as well as improve maternal and neonatal outcomes. In addition, due to the migration around the world, the pregnant women who come from the high prevalence area should screen for thalassemia, even if there is no policy in the neighborhood hospital. For example, the Asian couple in USA need to perform a thalassemia screening. This is a benefit for such couple whether they are a couple at risk for having a baby with major thalassemia diseases and prenatal diagnosis may be necessary.

## Conclusion

Although women who have thalassemia traits have no significant signs and symptoms, they will experience a higher rate of adverse pregnancy outcomes compared to pregnant women without thalassemia. Physiological changes in maternal circulation during pregnancy may decrease the hematocrit level and worsen the severity of anemia in the thalassemia trait group. Maternal anemia also affects pregnancy outcomes. Therefore, a high level of prenatal care and co-working between obstetricians and hematologists should be considered in thalassemia trait pregnant women to improve pregnancy outcomes and prevent adverse complications, as well as maintain a normal hematocrit level throughout pregnancy.
